# Supplementary figures and images for: CXCL1: A new diagnostic biomarker for human tuberculosis discovered using Diversity Outbred mice
Source: PLoS Pathog. 2021 Aug 17;17(8):e1009773. doi: 10.1371/journal.ppat.1009773 (PMC8423361; doi:10.1371/journal.ppat.1009773)

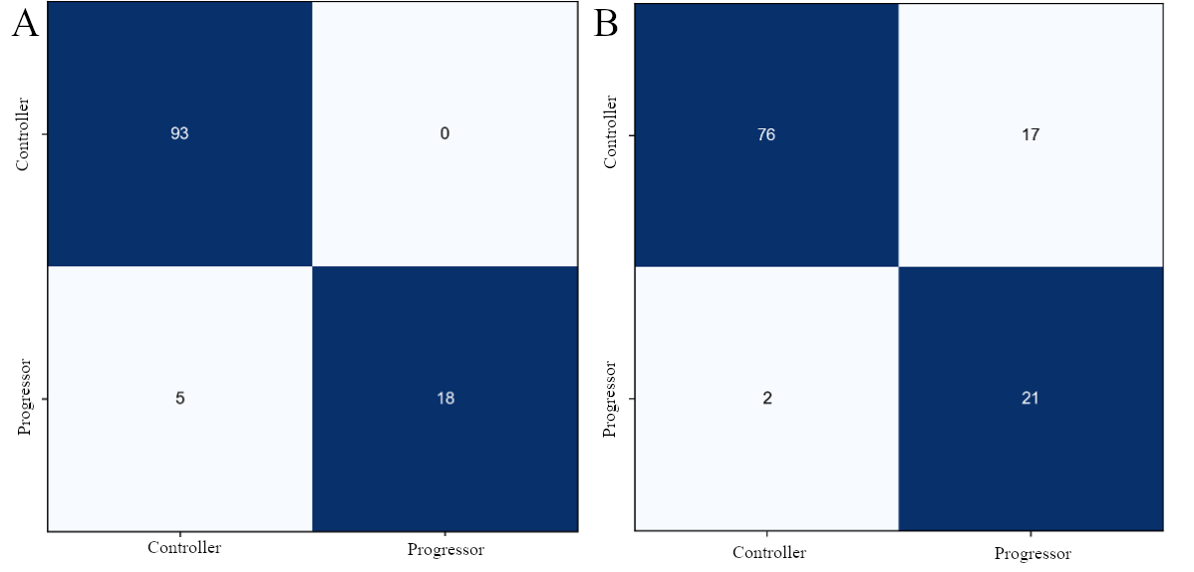

Supplement: S1 Fig — A) Logistic Regression with MMP8 and B) Gradient Tree Boosting with CXCL1, CXCL2, TNF, and IL-10. Row labels and column labels indicate the correct and the predicted labels, respectively. (TIF) [file ppat.1009773.s002.tif]

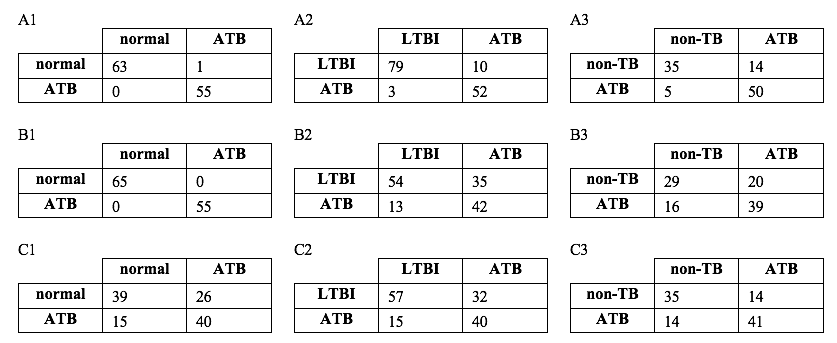

Supplement: S2 Fig — The rows in the overall figure correspond to CXCL1, MMP8, and S100A8, respectively. The first column denotes the results for ATB vs pooled normal samples, the second denotes ATB vs LTBI, and the last one denotes ATB vs non-TB. For each confusion matrix row labels and column labels indicate the correct and the predicted labels, respectively. (PNG) [file ppat.1009773.s003.png]

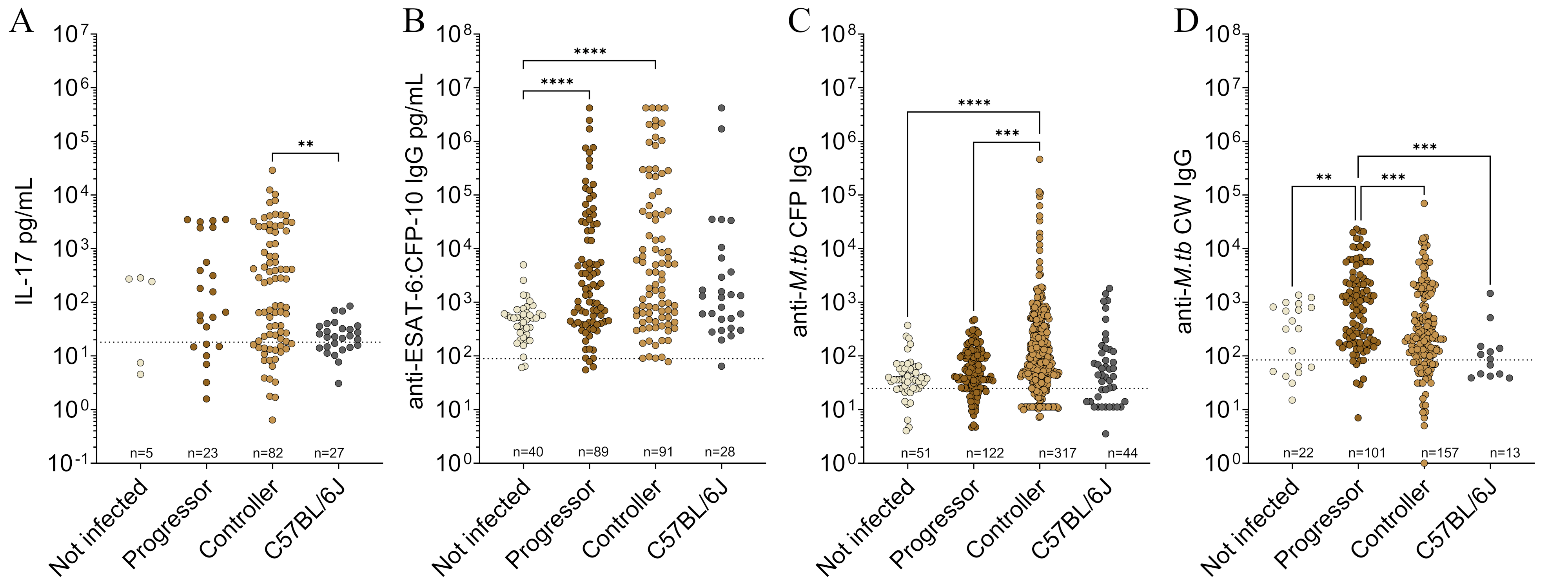

Supplement: S3 Fig — Additional lung proteins that we measured are shown (A-D). All data were lognormal distributed and analyzed by Kruskal-Wallis one-way ANOVA with Dunn’s multiple comparisons post-tests (*p<0.05; ***p<0.001). Each dot represents 1 mouse. Dashed lines show the limits of detection (LOD): For A-D LODs are 18.08 pg/mL, 89.27 pg/mL, 24.80 pg/mL, and 84.12 pg/mL, respectively. (TIF) [file ppat.1009773.s004.tif]
