# Supplementary material for: CXCL1: A new diagnostic biomarker for human tuberculosis discovered using Diversity Outbred mice
Source: PLoS Pathog. 2021 Aug 17;17(8):e1009773. doi: 10.1371/journal.ppat.1009773 (PMC8423361; doi:10.1371/journal.ppat.1009773)
Supplement: S1 Table — Enrichr identified the following gene ontology pathways using a set of 119 genes that were highly expressed in the lungs of progressor DO mice compared to non-infected DO mice and to controller DO mice. GO terms with adjusted p < 0.01 are shown, along with the human homologs of the genes overlapping with the term. Expressed genes in bold were pursued as diagnostic biomarkers. (DOCX) [file ppat.1009773.s005.docx]

| **Gene Ontology Term** | **GO Term ID** | **p** | **Adjusted p** | **Genes** |
| --- | --- | --- | --- | --- |
| cellular response to cytokine stimulus | GO:0071345 | 1.82E-13 | 9.28E-10 | CCR1, IL11, IL1RN, CSF3R, IL1R2, FPR1, OSM, F13A1, ACOD1, IL1RAP, **CXCL2**, MMP9, EREG, IL1A, IL6, IL1B, CCL4, CCL3, SAA1, PRTN3, TIMP1 |
| cytokine-mediated signaling pathway | GO:0019221 | 1.90E-13 | 4.86E-10 | CCR1, IL11, IL1RN, CSF3R, SERPINB2, RSAD2, IL1R2, FPR1, OSM, F13A1, IL1RAP, PPBP, CXCL3, **CXCL2**, MMP9, EREG, IL1A, IL6, IL1B, CCL4, CCL3, SAA1, PRTN3, TIMP1 |
| neutrophil mediated immunity | GO:0002446 | 6.46E-13 | 1.10E-09 | MGAM, ARG1, FPR1, PLAUR, PPBP, OLFM4, **MMP8**, ABCA13, ORM2, MMP9, IL6, SELL, TARM1, CXCR2, PRTN3, PGLYRP1, S100A9, **S100A8**, CAMP, CD177, LTF |
| neutrophil degranulation | GO:0043312 | 4.38E-12 | 5.59E-09 | MGAM, ARG1, FPR1, PLAUR, PPBP, OLFM4, **MMP8**, ABCA13, ORM2, MMP9, SELL, TARM1, CXCR2, PRTN3, PGLYRP1, S100A9, **S100A8**, CAMP, CD177, LTF |
| neutrophil activation involved in immune response | GO:0002283 | 5.09E-12 | 5.20E-09 | MGAM, ARG1, FPR1, PLAUR, PPBP, OLFM4, **MMP8**, ABCA13, ORM2, MMP9, SELL, TARM1, CXCR2, PRTN3, PGLYRP1, S100A9, **S100A8**, CAMP, CD177, LTF |
| neutrophil migration | GO:1990266 | 4.55E-11 | 3.87E-08 | CXCR2, CCL4, CCL3, SAA1, PRTN3, CXCL3, S100A9, **S100A8**, CD177 |
| positive regulation of leukocyte chemotaxis | GO:0002690 | 2.29E-09 | 1.67E-06 | CCR1, IL6, SERPINE1, CCL4, CCL3, PPBP, CXCL3, **CXCL2** |
| inflammatory response | GO:0006954 | 2.58E-09 | 1.64E-06 | FPR1, PPBP, CXCL3, **CXCL2**, IL1A, IL6, IL1B, CXCR2, CCL4, CCL3, PROK2, S100A9, **S100A8** |
| neutrophil chemotaxis | GO:0030593 | 2.27E-08 | 1.29E-05 | CXCR2, CCL4, CCL3, SAA1, CXCL3, S100A9, **S100A8** |
| granulocyte chemotaxis | GO:0071621 | 3.37E-08 | 1.72E-05 | CXCR2, CCL4, CCL3, SAA1, CXCL3, S100A9, **S100A8** |
| positive regulation of inflammatory response | GO:0050729 | 2.18E-07 | 1.01E-04 | IL6, SERPINE1, CCL4, CCL3, OSM, S100A9, **S100A8** |
| chemokine-mediated signaling pathway | GO:0070098 | 5.47E-07 | 2.33E-04 | CCR1, CCL4, CCL3, PPBP, CXCL3, **CXCL2** |
| extracellular matrix organization | GO:0030198 | 9.00E-07 | 3.53E-04 | COL1A1, ADAMTS4, VCAN, LOX, SERPINE1, TNC, SPP1, TIMP1, **MMP8**, MMP9 |
| defense response to bacterium | GO:0042742 | 1.43E-06 | 5.21E-04 | SELP, IL6, DMBT1, SERPINE1, REG3G, PGLYRP1, S100A9, **S100A8**, CAMP, LTF |
| cellular response to interleukin-1 | GO:0071347 | 2.66E-06 | 9.05E-04 | IL1A, IL1RN, IL1R2, IL1B, CCL4, CCL3, ACOD1, IL1RAP |
| acute-phase response | GO:0006953 | 3.09E-06 | 9.85E-04 | IL1RN, IL6, IL1B, SAA1 |
| positive regulation of defense response | GO:0031349 | 4.49E-06 | 1.35E-03 | SERPINE1, CCL4, CCL3, S100A9, **S100A8**, EREG |
| regulation of inflammatory response | GO:0050727 | 5.65E-06 | 1.60E-03 | SEMA7A, SERPINE1, CCL4, CCL3, SAA1, ACOD1, S100A9, **S100A8** |
| leukocyte aggregation | GO:0070486 | 6.54E-06 | 1.76E-03 | IL1B, S100A9, **S100A8** |
| antimicrobial humoral immune response mediated by antimicrobial peptide | GO:0061844 | 9.36E-06 | 2.39E-03 | REG3G, PGLYRP1, S100A9, CAMP, LTF |
| positive regulation of response to external stimulus | GO:0032103 | 1.40E-05 | 3.40E-03 | SERPINE1, CCL4, CCL3, ACOD1, S100A9, **S100A8** |
| response to molecule of bacterial origin | GO:0002237 | 2.28E-05 | 5.29E-03 | SELP, IL6, ACOD1, PPBP, CXCL3, **CXCL2** |
| regulation of leukocyte chemotaxis | GO:0002688 | 2.62E-05 | 5.81E-03 | IL6, PPBP, CXCL3, **CXCL2** |
| urea cycle | GO:0000050 | 3.03E-05 | 6.45E-03 | ARG2, ARG1, ACOD1 |
| response to lipopolysaccharide | GO:0032496 | 3.37E-05 | 6.88E-03 | SELP, IL6, SERPINE1, ACOD1, PPBP, CXCL3, **CXCL2** |
| positive regulation of cell proliferation | GO:0008284 | 3.65E-05 | 7.16E-03 | IL11, IL6, IL1B, CXCR2, OSM, PROK2, PRTN3, REG3G, TIMP1, EREG, LTF |
| defense response to Gram-positive bacterium | GO:0050830 | 3.77E-05 | 7.12E-03 | IL6, DMBT1, REG3G, PGLYRP1, CAMP |
| positive regulation of leukocyte migration | GO:0002687 | 4.36E-05 | 7.94E-03 | IL6, PPBP, CXCL3, **CXCL2** |
| positive regulation of cellular process | GO:0048522 | 4.89E-05 | 8.60E-03 | IL11, IL6, IL1B, CXCR2, OSM, PROK2, PRTN3, REG3G, TIMP1, S100A9, **S100A8**, EREG |
| negative regulation of hormone secretion | GO:0046888 | 5.21E-05 | 8.86E-03 | IL11, IL1B, OSM |
| defense response to Gram-negative bacterium | GO:0050829 | 5.77E-05 | 9.51E-03 | SELP, IL6, DMBT1, SERPINE1, LTF |
