# Supplementary material for: CXCL1: A new diagnostic biomarker for human tuberculosis discovered using Diversity Outbred mice
Source: PLoS Pathog. 2021 Aug 17;17(8):e1009773. doi: 10.1371/journal.ppat.1009773 (PMC8423361; doi:10.1371/journal.ppat.1009773)
Supplement: S2 Table — The first three rows achieved the highest AUC and the remaining rows achieved comparable AUC with a simpler model. "Threshold" denotes the probability threshold selected. The standard deviation of the five folds is denoted with ±. (DOCX) [file ppat.1009773.s006.docx]

| **Panel** | **Algorithm** | **AUC** | **Specificity** | **Sensitivity** | **Threshold** |
| --- | --- | --- | --- | --- | --- |
| CXCL1, IL-12, MMP8, VEGF, S100A8 | Gradient Tree Boosting | 0.98 ± 0.02 | 88.9 ± 4.0 | 93.1 ± 6.2 | 0.49869 |
| CXCL1, MMP8 | Gradient Tree Boosting | 0.97 ± 0.01 | 86.0 ± 9.2 | 88.9 ± 7.8 | 0.49869 |
| CXCL1, IL-12, MMP8 | Gradient Tree Boosting | 0.97 ± 0.01 | 85.8 ± 1.1 | 89.9 ± 7.1 | 0.48535 |
| CXCL2 | Logistic Regression | 0.95 ± 0.02 | 83.1 ± 22.8 | 89.9 ± 7.4 | 0.31535 |
| CXCL1 | Logistic Regression | 0.96 ± 0.01 | 75.4 ± 7.6 | 95.5 ± 4.2 | 0.19525 |
| MMP8 | Logistic Regression | 0.95 ± 0.03 | 87.4 ± 6.0 | 94.1 ± 3.1 | 0.19111 |
