# Supplementary material for: CXCL1: A new diagnostic biomarker for human tuberculosis discovered using Diversity Outbred mice
Source: PLoS Pathog. 2021 Aug 17;17(8):e1009773. doi: 10.1371/journal.ppat.1009773 (PMC8423361; doi:10.1371/journal.ppat.1009773)
Supplement: S3 Table — Columns under “Leave-one-exp-out” and “Four-exp-combined” correspond to the performance in leave-one-experiment-out setting and four-experiments-combined setting, respectively. “Spec.” denotes specificity and “Sens.” denotes sensitivity. Sensitivity (specificity) under the “Exp-Wise” columns indicate experiment-wise sensitivity (specificity) and sensitivity (specificity) under the “Min. Exp.” columns indicate minimum experiment sensitivity (specificity) and sensitivity (specificity) under the “Overall” columns indicate sensitivity (specificity) (As defined in Materials and methods). (DOCX) [file ppat.1009773.s007.docx]

| **Panel** | **Algorithm** | **Leave-one-exp-out** | | | | **Four-exp-combined** | | | | | |
| --- | --- | --- | --- | --- | --- | --- | --- | --- | --- | --- | --- |
|  |  | **Exp-Wise** | | **Min. Exp.** | | **Overall** | | **Exp-Wise** | | **Min. Exp.** | |
|  |  | **Spec.** | **Sens.** | **Spec.** | **Sens.** | **Spec.** | **Sens.** | **Spec.** | **Sens.** | **Spec.** | **Sens.** |
| CXCL2, CXCL1, TNF,  IL-10 | Gradient Tree Boosting | 88.5 | 88.7 | 78.8 | 83.3 | 88.8 | 96.6 | 83.1 | 97.3 | 75.8 | 93.3 |
| CXCL2, CXCL1, IL-10 | Gradient Tree Boosting | 87.6 | 94.8 | 78.8 | 83.3 | 91.5 | 94.3 | 86.2 | 95.5 | 75.8 | 90.0 |
| CXCL2, TNF, IL-12,  IL-10 | Gradient Tree Boosting | 83.7 | 92.9 | 72.7 | 83.3 | 88.4 | 93.2 | 81.4 | 94.5 | 71.4 | 90.0 |
