# Supplementary material for: CXCL1: A new diagnostic biomarker for human tuberculosis discovered using Diversity Outbred mice
Source: PLoS Pathog. 2021 Aug 17;17(8):e1009773. doi: 10.1371/journal.ppat.1009773 (PMC8423361; doi:10.1371/journal.ppat.1009773)
Supplement: S4 Table — "Avg." is average and "Diff." is difference. In Materials and Methods, the calculation for average % difference is described. To calculate the average difference, the same method is used but instead of averaging over % difference, it is averaged over difference. Average AUC denotes the average AUC of the biomarker panels that contain the biomarker. Average top 5 denotes the average AUC of the five biomarker panels with highest AUC that contain the biomarker and average bottom 5 denotes the average AUC of the five biomarker panels with the least AUC that contain the biomarker. (DOCX) [file ppat.1009773.s008.docx]

| **Biomarker** | **Avg. % Diff.** | **Avg. Diff.** | **Avg. AUC** | **Avg. Top 5** | **Avg. Bottom 5** |
| --- | --- | --- | --- | --- | --- |
| MMP8 | 11.31 | 0.08 | 0.95 | 0.96 | 0.95 |
| CXCL1 | 5.8 | 0.04 | 0.95 | 0.96 | 0.92 |
| CXCL2 | 4.82 | 0.03 | 0.94 | 0.96 | 0.91 |
| TNF | 3.37 | 0.02 | 0.94 | 0.95 | 0.93 |
| S100A8 | 2.72 | 0.02 | 0.92 | 0.96 | 0.77 |
| CXCL5 | 2.25 | 0.01 | 0.94 | 0.95 | 0.89 |
| IFN-g | 1.14 | 0.01 | 0.93 | 0.95 | 0.83 |
| IL-10 | 0.04 | 0 | 0.92 | 0.96 | 0.67 |
| IL-12 | -0.35 | 0 | 0.92 | 0.96 | 0.65 |
| VEGF | -1.29 | -0.01 | 0.91 | 0.96 | 0.64 |
