# Supplementary material for: CXCL1: A new diagnostic biomarker for human tuberculosis discovered using Diversity Outbred mice
Source: PLoS Pathog. 2021 Aug 17;17(8):e1009773. doi: 10.1371/journal.ppat.1009773 (PMC8423361; doi:10.1371/journal.ppat.1009773)
Supplement: S5 Table — 95% confidence intervals are denoted in the parenthesis. AUC values with the confidence intervals were calculated using pROC [50]. (DOCX) [file ppat.1009773.s009.docx]

| **Biomarker** | **AUC** |
| --- | --- |
| CXCL5 | 0.89 (0.86-0.93) |
| CXCL2 | 0.96 (0.94-0.99) |
| CXCL1 | 0.97 (0.95-0.98) |
| IFN-g | 0.81 (0.76-0.86) |
| TNF | 0.92 (0.89-0.95) |
| IL-12 | 0.61 (0.55-0.67) |
| IL-10 | 0.66 (0.6-0.71) |
| MMP8 | 0.96 (0.93-0.99) |
| VEGF | 0.58 (0.51-0.65) |
| S100A8 | 0.84 (0.79-0.89) |
