# Supplementary material for: CXCL1: A new diagnostic biomarker for human tuberculosis discovered using Diversity Outbred mice
Source: PLoS Pathog. 2021 Aug 17;17(8):e1009773. doi: 10.1371/journal.ppat.1009773 (PMC8423361; doi:10.1371/journal.ppat.1009773)
Supplement: S6 Table — Patients that are missing demographic information are omitted from the results displayed. For rows next to “Country” and “Sex” the number of patients in each category and its percentage is given. At the final row, the median and the IQR of the age is given. (DOCX) [file ppat.1009773.s010.docx]

| FIND  (Geneva, Switzerland) | Country | Vietnam | 138 (73.8%) |
| --- | --- | --- | --- |
|  |  | South Africa | 49 (26.2%) |
|  | Sex | Male | 125 (66.8%) |
|  |  | Female | 62 (33.2%) |
|  | Median Age (IQR) | | 55 (30.0) |
