# Supplementary material for: CXCL1: A new diagnostic biomarker for human tuberculosis discovered using Diversity Outbred mice
Source: PLoS Pathog. 2021 Aug 17;17(8):e1009773. doi: 10.1371/journal.ppat.1009773 (PMC8423361; doi:10.1371/journal.ppat.1009773)
Supplement: S1 File — (DOCX) [file ppat.1009773.s013.docx]

**Supplementary Materials for**

CXCL1: A new diagnostic biomarker for human tuberculosis discovered using Diversity Outbred mice.

**Authors:** Deniz Koyuncu, Muhammad Khalid Khan Niazi, Thomas Tavolara, Claudia Abeijon, Melanie L. Ginese, Yanghui Liao, Carolyn Mark, Aubrey Specht, Adam C. Gower, Blanca I. Restrepo, Daniel M. Gatti, Igor Kramnik, Metin Gurcan, Bülent Yener, Gillian Beamer

Includes the following:

Supplementary Methods

Supplementary Text

Supplementary References

**Supplementary Methods**

**Sensitivity, Specificity and AUC**

Sensitivity is the ratio of the correctly diagnosed positive samples to the total number of positive samples. Specificity is the ratio of the negative samples that were correctly diagnosed to the total number of negative samples. As an example, the requirement for a triage test is at least 90% sensitivity and 70% specificity which requires at least 90% of the positive samples and 70% of the negative samples to be correctly diagnosed.

To calculate AUC, first an ROC curve is drawn. To draw an ROC curve, we first vary the threshold of the classifier and re-diagnose the samples for each threshold. For each threshold, we draw a point on the Cartesian coordinate plane whose x coordinate equals to 1-specificity and y coordinate is equals to the sensitivity. Connecting the dots will result in a monotonically increasing curve which as an example Fig 5 can be given. AUC is the area under the resulting curve which can be computed using a numerical integral. AUC is between 0 and 1. Generally a higher AUC is preferable where AUC equals to 1 indicates 100% sensitivity and specificity can be achieved. The AUC values with the confidence intervals in the S5 Table are calculated using pROC [1].

**Selecting the representative classification algorithms**

To simplify analyses, we selected one-representative algorithm from linear and non-linear classification algorithms after observing similarity in performance. One factor that we considered was the support for estimating probability values since we were interested in AUC analyses. SVM Linear and SVM RBF by default do not support estimating probabilities and were not pursued. Gradient Tree Boosting and Random Forest are more suitable because of the probabilistic formulation of their loss function. Therefore, after initial study, we decided to not pursue SVM. Gradient Tree Boosting and Random Forest both use an ensemble of decision trees but Gradient Tree Boosting which uses gradient boosting is empirically shown to have a higher performance [2]. Thus, we selected Gradient Tree Boosting as the non-linear classification algorithm.

The first selection Logistic Regression with L1-regularization has a linear decision boundary with L1-regularization and relatively resistant to overfitting. The second selection Gradient Tree Boosting is an ensemble method that achieves competitive performance in various benchmark datasets [2] and has been previously used in TB biomarker studies as well [3,4].

**Classification algorithm, hyper-parameter, and feature selection details**

In the first approach, for further analysis, three classifiers with the highest AUC are selected and three classifiers that use a linear decision boundary and a single biomarker which achieved comparable AUCs are selected (S2 Table). Operating points of the selected classifiers are tuned to achieve >90% sensitivity and >70% specificity. Of those, we selected the classifier with the highest sensitivity whose specificity is >70% specificity after subtracting one standard deviation.

In the second approach, after selecting the hyper-parameters of the classifiers, for each classifier the operating point that maximizes the experiment-wise sensitivity while achieving at least 70% specificity in each of the experiments is selected. Afterward, we identified the classifiers that achieved both minimum experiment sensitivity ≥90% and specificity ≥70 in the four-experiments combined setting and minimum experiment sensitivity ≥80% and specificity ≥70% in the leave-one-experiment-out setting (S3 Table). Among the classifiers identified the one with the highest minimum experiment sensitivity (93.3%) is selected.

Feature rankings

In the initial classifier selection, we have already measured the AUC of the 239 biomarker combinations using 5-fold-CV on the training portion of the discovery cohort. To calculate the average percent improvement for a biomarker, first, a subset of the 239 biomarker combinations that includes that biomarker are identified. Then, for each identified biomarker combination, the percent difference between the AUC of that combination and the AUC of the biomarker combination without that biomarker is calculated. Finally, percent differences calculated for each of the identified combinations are averaged to calculate the average percent difference for that biomarker. As the AUC of a biomarker combination, we have used the AUC of the Logistic Regression with L1 regularization and the hyper-parameter with the highest AUC is used.

Feature rankings plot

The same methodology is used for the initial classifier selection with the exception that all 1023 combinations of the 10 biomarkers are searched and (0.01, 0.3, 0.5,1.) are searched for the hyper-parameters of Gradient Tree Boosting.

**Logistic Regression with L1 Regularization**

We have used the Scikit-learn [5] implementation of Logistic Regression with L1 Regularization whose mathematical description is given below. Let $\boldsymbol{x}_{i}\in R^{D}$ denote the feature vector and $y_{i}\in\{-1,1\}$ as the label of i’th sample respectively where D is the number of biomarkers in the panel and $\{-1,1\}$ denote controller and progressor, respectively. Let $\boldsymbol{w}\in R^{D}$denote the weights of the features and $b\in R$the bias term, the optimal parameters are found by optimizing the following problem.

$$\boldsymbol{w}^{*},b^{*}=\arg\min_{\mathbf{w},b} \sum_{i=1}^{N} \log(1+e^{-y_{i}\left( \boldsymbol{w}^{T}\boldsymbol{x}_{i}+b \right)})+\lambda\left\| \boldsymbol{w} \right\|_{1}$$

where $\left\| . \right\|_{1}$ denotes the L1 norm.

We have treated $\lambda$ as a hyper-parameter and applied grid search to select it as described in Materials and Methods.

Gradient Tree Boosting

Gradient boosting is a way of combining a set of weak learners. Each stage a weak learner is fit to the “pseudo-residuals” resulting from combinations of the weak learners so far. After fitting the weak learner, its prediction function is added to the combined prediction function after multiplied by a shrinkage factor [6].

Gradient Tree Boosting uses regression trees as weak learners and uses a slight variation of gradient boosting algorithm where after fitting the regression tree to the “pseudo-residuals” the prediction value for each region is updated again [6]. The algorithm without the shrinkage is given in Algorithm 10.3 in [6].

We have treated maximum depth of the trees, the number of weak learners (referred to as the number of trees), and the shrinkage factor (referred to as the learning rate) as hyper-parameters and selected them through grid search as described in Materials and Methods. The remaining hyper-parameters are used as the default values provided in Scikit-learn [5].

**Supplementary Text**

**Selection of the first classifier.**

Three classifiers achieved the highest area under the receiver operating characteristics curve (AUC) values: Gradient Tree Boosting with CXCL1, IL-12, MMP8, VEGF, and S100A8; Gradient Tree Boosting with CXCL1 and MMP8; and Gradient Tree Boosting with CXCL1, IL-12, and MMP8. The other three classifiers had comparable AUC values and used a single biomarker with a linear decision boundary: Logistic Regression with CXCL2, CXCL1, or MMP8 (S2 Table). All six classifiers performed well, with AUCs between 0.95 and 0.98. To refine further, we tuned the operating points to meet the WHO TPP triage test specifications (>90% sensitivity and >70% specificity): Logistic Regression with CXCL1; Logistic Regression with MMP8; and Gradient Tree Boosting with CXCL1, IL-12, MMP8, VEGF, and S100A8. Of those, we selected Logistic Regression with MMP8 for validation, because its sensitivity (94.1%) is higher than that of the Gradient Tree Boosting classifier with five proteins, while its specificity is comparable (87.4%).

**Selection of the second classifier.**

In the four-experiment combined setting, 21 out of 478 classifiers achieve minimum experiment sensitivity ≥90 and specificity ≥70. In the leave-one-experiment-out task, 11 out of the 478 classifiers achieved minimum testing sensitivity ≥80% and specificity ≥70%. None of them achieved higher than 90% minimum testing sensitivity. Three classifiers that satisfy both criteria are listed in (S3 Table). We have observed that the number of classifiers achieved sensitivity ≥90 and specificity ≥70 in each of the experiments is less than to the number of classifiers that have experiment wise sensitivity ≥90 and specificity ≥70. We have also observed that the number of classifiers that achieved overall sensitivity ≥90 and specificity ≥70 is higher than the number of classifiers that achieved the same values using experiment-wise averages.

**Within Experiment Performance of the Selected Classifiers.**

Logistic regression with MMP8 is evaluated in the testing portion of the discovery cohort in four-experiments combined setting. It achieved sensitivity values within Experiments 1-4, 100%, 100%, 100%, and 89%, respectively and specificity values are 40%, 100%, 70%, and 95% respectively.

In the testing portion of the discovery cohort, Gradient Tree Boosting with CXCL1, CXCL2, TNF, and IL-10 is evaluated in four-experiments combined setting. Its sensitivity values within Experiments 1-4 were 87.5%, 100%, 100%, and 100%, respectively, with specificities of 77.8%, 100%, 100%, and 100%, respectively.

**Supplementary References:**

1. Robin X, Turck N, Hainard A, Tiberti N, Lisacek F, Sanchez J-C, et al. pROC: an open-source package for R and S+ to analyze and compare ROC curves. BMC Bioinformatics. 2011;12(1):77. doi: 10.1186/1471-2105-12-77.

2. Olson RS, La Cava W, Mustahsan Z, Varik A, Moore JH. Data-driven Advice for Applying Machine Learning to Bioinformatics Problems. arXiv:170805070 [cs, q-bio, stat]. 2018.

3. Ahmad R, Xie L, Pyle M, Suarez MF, Broger T, Steinberg D, et al. A rapid triage test for active pulmonary tuberculosis in adult patients with persistent cough. Sci Transl Med. 2019;11(515). Epub 2019/10/28. doi: 10.1126/scitranslmed.aaw8287. PubMed PMID: 31645455.

4. Leong S, Zhao Y, Ribeiro-Rodrigues R, Jones-López EC, Acuña-Villaorduña C, Rodrigues PM, et al. Cross-validation of existing signatures and derivation of a novel 29-gene transcriptomic signature predictive of progression to TB in a Brazilian cohort of household contacts of pulmonary TB. Tuberculosis. 2020;120:101898. doi: 10.1016/j.tube.2020.101898.

5. Pedregosa F, Varoquaux G, Gramfort A, Michel V, Thirion B, Grisel O, et al. Scikit-learn: Machine Learning in Python. Journal of Machine Learning Research. 2011;12:2825--30.

6. Hastie T, Tibshirani R, Friedman J. The Elements of Statistical Learning: Data Mining, Inference, and Prediction. New York City, USA: Springer; 2009.
