# Supplementary material for: CXCL1: A new diagnostic biomarker for human tuberculosis discovered using Diversity Outbred mice
Source: PLoS Pathog. 2021 Aug 17;17(8):e1009773. doi: 10.1371/journal.ppat.1009773 (PMC8423361; doi:10.1371/journal.ppat.1009773)
Supplement: Attachment — Submitted filename: response_letter_PPATHOGENS-D-21-00542.pdf [file ppat.1009773.s014.pdf]

Tufts UNIVERSITY | Cummings School of Veterinary Medicine

10 June 2021

Dear PLOS Pathogens,

We are grateful for the positive responses to PPATHOGENS-D-21-00542. Changes to the original manuscript are blue text in the revised manuscript. Based on the reviews, we made 5 substantive changes:

1. Changed DO mouse terms from "supersusceptible" and "not-supersusceptible" to *progressor* and *controller*. This change aligns with Ahmed *et al* Sci Trans Medicine 2020, Jan 29; 12 (528) "Immune correlates of tuberculosis disease and risk translate across species."
2. Tested an additional biomarker candidate on human serum samples (e.g., S100A8)
3. Analyzed additional human patient samples (e.g., non-tuberculous lung disease).
4. Based on results from 2&3, revised the title to emphasize that serum CXCL1 discriminated ATB patients from important differential diagnoses: LTBI and non-TB lung disease.
5. Added 2 authors: Dr Blanca Restrepo, for critical insight and analysis of human data; and Ms Aubrey Specht, for critical analysis of mouse data. Ms Specht's omission in the original submission was an oversight that we are happy to correct here.

Detailed responses to all Major & Minor issues are below:

Major Issues: Key Experiments Required for Acceptance

Reviewer #1:

What timepoint post-infection the microarrays were performed (Figure 2).

- All *progressors* euthanasia timepoints were 21-60 days. *Controllers* euthanasia timepoints were between 95-157 days. Eleven *controllers* met morbidity criteria between 95-157 days; and 11 *controllers* survived 157 days without morbidity and were euthanized on day 157. See Figure 2 caption.

If the samples were taken at different times post-infection, does this confound the analysis?

- We do not think so because we are comparing different disease state across genotypes, not disease progression within fixed genotypes.

Is there a way to match timepoints for "supersusceptible" and "Not supersusceptible" mice?

- Not quite yet. The challenge is: We do not have a means to distinguish *progressor* and *controller* individuals (to match timepoints of pairs) prospectively. We are working on machine-learning models to find genotype-predictions to be able to do so.

Would it be appropriate to include C57BL/6 mice at the same timepoints as "supersusceptible" mice?

- Opinions differ whether inbred mice are good controls for outbred DOs. There is no definite right answer. We included C57BL/6J mice at the peak (5 weeks) and end (8 weeks) of the *progressor* mortality wave in the lung protein measurements (Fig 3), and downstream analyses. See Table 1 and Figure 3 caption.

Include fold-change values for *S100a8, Mmp8* and *Cxcl2* from the microarray analysis; include discussion of why these 3 genes were pulled out, and if there are other genes that could be analyzed in the future.

- Explanations are in lines 147-149. Briefly, pulled out due to significantly increased expression, prior study (Niazi 2015), and reagent availability. Significances and fold changes are now included in lines 136-139 and Figure 2 caption.

- Other genes that could be analyzed in the future are now published in Tavolara, *et al.* EBioMedicine Volume 67, May 2021, 103388 "Deep learning predicts gene expression as an intermediate data modality to identify susceptibility patterns in *Mycobacterium tuberculosis*-infected Diversity Outbred mice." This paper identified 5 highly, differentially expressed genes capable of classifying *progressor* mice.

Reviewer #2:

Clarify the machine learning descriptions. For example, "classifier" is vague.
- We agree. We defined the term after its first occurrence, line 170.

Comment on why Logistic Regression and Gradient Tree Boosting were selected and Support Vector Machine, SVM with Radial Basis Function, and Random Forest were not selected.
- We explained the reasoning in lines 473-478 of the manuscript and details in Supplementary methods lines 805-819.

Explain how the study design shown in Fig. 4 facilitates these approaches.
- We explained the purpose in lines 432-435; 461-469. Briefly, Figure 4 shows how data sets were divided into training, validation, and independent testing cohorts for machine learning.

Include the meaning and significance of sensitivity, specificity, and AUC statistics. Consider moving ROC curves (Fig. S1) and referencing the confusion matrices (Fig. S2 & Fig. S3).
- We defined the metrics in Supplementary Methods, lines 789-801, and better interpreted the meaning in context of patient diagnoses in lines 65-68 (introduction) and lines 283-287 (discussion). We moved the ROC curves Figure 5 and referenced the confusion matrices in lines 178 and 194.

Reviewer #3:

Examine whether human TB biomarkers except IFN-gamma (e.g. C-reactive protein, transferrin, etc…) show differential expression in the DO study.
- We examined the microarray data for those TB biomarkers. None are highly differentially expressed upregulated > 2-fold in progressor DO mouse lungs relative to non-infected or controller mice. The differences may be attributable to the tissue samples (blood vs lungs and the cellular content therein).

Part III – Minor Issues: Editorial and Data Presentation Modifications

Reviewer #1:

Clarify "supersusceptible" vs "Not supersusceptible."
- Throughout the manuscript, we do not use supersusceptible and not-supersusceptible. The revised manuscript uses *progressor* and *controller* DO mice aligning with Ahmed *et al* Sci Trans Medicine 2020, Jan 29; 12 (528) "Immune correlates of tuberculosis disease and risk translate across species."
- Materials and Methods lines 371-374, Table 1, and Figure 3 caption contain the same information.

Address the paper from Kathamuthu et. al "Matrix Metalloproteinases and Tissue Inhibitors of Metalloproteinases Are Potential Biomarkers of Pulmonary and Extra-Pulmonary Tuberculosis" Front Immunology (2020) in the discussion.
- Thank you. This is a great idea. We have added to the discussion lines 293-297.

Reviewer #2:

Truncation of the survival data in Fig. 1 undercuts the human-like spectrum of phenotypes across the DO population. Would a longer survival curve better illustrate the groups?

- Yes, we agree, but our survival studies are not finished yet. We will publish them elsewhere. For other long-term studies, the reviewer is refered to Kurtz *et al.* "The Diversity Outbred Mouse Population is an Improved Animal Model of Vaccination against Tuberculosis That Reflects Heterogeneity of Protection." mSphere. 2020 Apr 15;5(2):e00097-20. doi: 10.1128/mSphere.00097-20

Explain the choice of Kruskal-Wallis one-way ANOVA. This assumes similar group distribution across tested groups, an assumption which does not seem to be met in some of the Fig. 3 subfigures (B, C, K, L).

- We consulted our statistician (Dr. Daniel Gatti, author) and searched for alternative suitable tests. None known to Dr Gatti met the exact structure of our data, and he also thinks Kruskal-Wallis one-way ANOVA is reasonable.

Studies do not all include B6 mice, which begs the question of how (or if) batch normalization was implemented for Exp. 1 & Exp. 3 (as per Fig. 4), especially given that batch variation is noted on line 211.

- The reviewer is correct. Exp. 1 & 3 used only DO mice. Exp 2, 4, 5 included C57BL/6J mice, and we used them all. We did not normalize batches prior to implementation. Instead, we applied a standardization technique (described in Preprocessing line 454-455).

The statement regarding "this is the first study" to use DO mice for translational biomarker discovery, this does not seem accurate. Review, cite, and discuss Gopal *et al.*, Am J Respir Crit Care Med (2013), in which S100A8 & S100A9 are examined as biomarkers of TB in DO mice; and Ahmed *et al.* Sci Transl Med (2020) DO mice, NHP and human cohorts.

- We softened the language. We reviewed, cited, and discussed Gopal *et al*. Authors identified statistically significant differences but did not evaluate diagnostic performance of S100A8/S100A9 complex (calprotectin), or individual components S100A8 (calgranulin A) and S100A9 (calgranulin B).
- Extending their work, we performed additional testing of new human patient data and analyses, showing that S100A8 does not met TPP diagnostic criteria. The revisions are lines 87-89; 100-101; 224-225; 228-230; 233-234; 307-312; Table 2; Figure 7.
- We reviewed, cited, and discussed Ahmed *et al.* As a result, we updated our original terminology (supersusceptible and not-supersusceptible) to match *progressor* and *controller*. We examined the publication, and analyzed their publicly available data, to see whether our 11 proteins overlapped their gene expression sets from mouse & macaque lungs, and human blood. The discussion has been expanded in lines 316-331.

Explain why both CXCL1 and CXCL2 are highlighted in Fig. 5 while the two other members of the four-biomarker panel (TNF, IL-10) are not. Consider changing the color scheme to emphasize classifiers with CXCL1 and MMP8 might better align with the other results.

- MMP8, CXCL1, and CXCL2 are highlighted because those three biomarkers contributed to the strong performance in DO mouse lungs (see Table S4). TNF and IL-10 were identified by feature selection algorithms but their contribution to performance was marginal.

Show and explain all statistically significant differences for the human samples, and any error bars. The variance on the ATB group looks like this group should not be significantly different from LTBI.

- Good idea. Figure 7 has been revised as suggested and shown as dot plots instead of bar graphs. Sample numbers are also included.

List abbreviations in the figure caption, so the figures stand alone

- Good idea. This is done for all figures in the revised manuscript.

Correct copyedits:
Line 90: "One third" = "One-third"
Fig. 1 Legend: "succumned" = "succumbed"
Fig. 2 Title: "unniquely" = "uniquely"

Line 192: "specify" = "specificity"
Line 324: "satisfies" = "satisfy"
Line 335: "than" = "then"
Line 597: Double space after "Enrichr"
Line 749: "leave-one-experiment" = "leave-one-experiment-out"

Thank you, we have corrected them.

Reviewer #3:

Provide DO generation information and whether information is available on kinship.

- Generation information is included in line 365. We order non-siblings from the Jackson Laboratory. Jax does not provide information on kinship.

Provide timepoints of euthanasia, mouse numbers, and detail throughout the manuscript results.

- We clarified the timepoints of euthanasia and mouse numbers by including them in Figure 2 and included a new table (Table 1) that denotes the duration of infection. In the Materials and Methods lines 371-374, we added the same information.

Sincerely,

Gillian Beamer, VMD, PhD, DACVP
